# Interfering with the AKT/mTOR/STAT3/ID1 signaling axis with usenamine A restrains the proliferative and invasive potential of human hepatocellular carcinoma cells

**DOI:** 10.1186/s13020-023-00875-w

**Published:** 2024-01-05

**Authors:** Ailin Yang, Huiming Huang, Jinxin Xie, Yingying Tian, Longyan Wang, Dongxiao Liu, Xuejiao Wei, Peng Tan, Xingyun Chai, Xiaojun Zha, Pengfei Tu, Zhongdong Hu

**Affiliations:** 1 Modern Research Center for Traditional Chinese Medicine, Beijing Research Institute of Chinese Medicine, Beijing University of Chinese Medicine, No. 11 North 3rd Ring East Road, Chaoyang District, Beijing, 100029 People’s Republic of China; 2 School of Pharmacy, Binzhou Medical University, Yantai, 264003 China; 3 Department of Biochemistry & Molecular Biology, School of Basic Medicine, Anhui Medical University, Hefei, 230032 China

**Keywords:** Usenamine A, Hepatocellular carcinoma, AKT/mTOR/STAT3/ID1 axis, Proliferation, Invasion

## Abstract

**Background:**

Usenamine A, a novel natural compound initially isolated from the lichen *Usnea longissima*, has exhibited promising efficacy against hepatoma in prior investigation. Nevertheless, the underlying mechanisms responsible for its antihepatoma effects remain unclear. Furthermore, the role of the AKT/mechanistic target of the rapamycin (mTOR)/signal transducer and activator of transcription 3 (STAT3)/inhibitor of differentiation/DNA binding 1 (ID1) signaling axis in hepatocellular carcinoma (HCC), and the potential anti-HCC effects of drugs targeting this pathway are not well understood.

**Methods:**

CCK-8 assay was used to investigate the effects of usenamine A on the proliferation of human HCC cells. Moreover, the effects of usenamine A on the invasion ability of human HCC cells were evaluated by transwell assay. In addition, expression profiling analysis, quantitative real-time PCR, immunoblotting, immunohistochemistry (IHC) analysis, RNAi, immunoprecipitation, and chromatin immunoprecipitation (ChIP) assay were used to explore the effects of usenamine A on the newly identified AKT/mTOR/STAT3/ID1 signaling axis in human HCC cells.

**Results:**

Usenamine A inhibited the proliferation and invasion of human HCC cell lines (HepG2 and SK-HEP-1). Through the analysis of gene expression profiling, we identified that usenamine A suppressed the expression of ID1 in human HCC cells. Furthermore, immunoprecipitation experiments revealed that usenamine A facilitated the degradation of the ID1 protein via the ubiquitin–proteasome pathway. Moreover, usenamine A inhibited the activity of STAT3 in human HCC cells. ChIP analysis demonstrated that STAT3 positively regulated ID1 expression at the transcriptional level in human HCC cells. The STAT3/ID1 axis played a role in mediating the anti-proliferative and anti-invasive impacts of usenamine A on human HCC cells. Additionally, usenamine A suppressed the STAT3/ID1 axis through AKT/mTOR signaling in human HCC cells.

**Conclusion:**

Usenamine A displayed robust anti-HCC potential, partly attributed to its capacity to downregulate the AKT/mTOR/STAT3/ID1 signaling pathway and promote ubiquitin–proteasome-mediated ID1 degradation. Usenamine A has the potential to be developed as a therapeutic agent for HCC cases characterized by abnormal AKT/mTOR/STAT3/ID1 signaling, and targeting the AKT/mTOR/STAT3 signaling pathway may be a viable option for treating patients with HCC exhibiting elevated ID1 expression.

## Background

Hepatocellular carcinoma (HCC) stands as a prominent cause of cancer-related fatalities worldwide, presenting substantial challenges in terms of prevention and treatment. Therefore, there exists an urgent necessity to explore potential therapeutics for HCC [1, 2].

The AKT/mechanistic target of the rapamycin (mTOR) pathway is frequency dysregulated in liver cancer and plays a critical role in cell proliferation and metastasis. It has emerged as an attractive therapeutic target for liver cancer [3, 4]. As a key transcription factor, signal transducer and activator of transcription 3 (STAT3) has a significant effect on tumor cell proliferation and metastasis [5]. STAT3 is constitutively activated in HCC and represents a potential therapeutic target for anti-HCC treatments [6]. Moreover, STAT3 has been identified as the downstream target of the AKT/mTOR pathway [7]. Inhibitor of differentiation/DNA binding 1 (ID1), a member of the helix-loop-helix protein family, has been documented to have pro-oncogenic properties, encouraging both proliferation and cancer metastasis [8]. Additionally, ID1 has been found to be overexpressed in HCC [9]. However, the role of the AKT/mTOR/STAT3/ID1 axis in liver cancer remains unexplored, and the effects of therapeutics targeting the AKT/mTOR/STAT3/ID1 signaling pathway remain unknown.

**Fig. 1:**
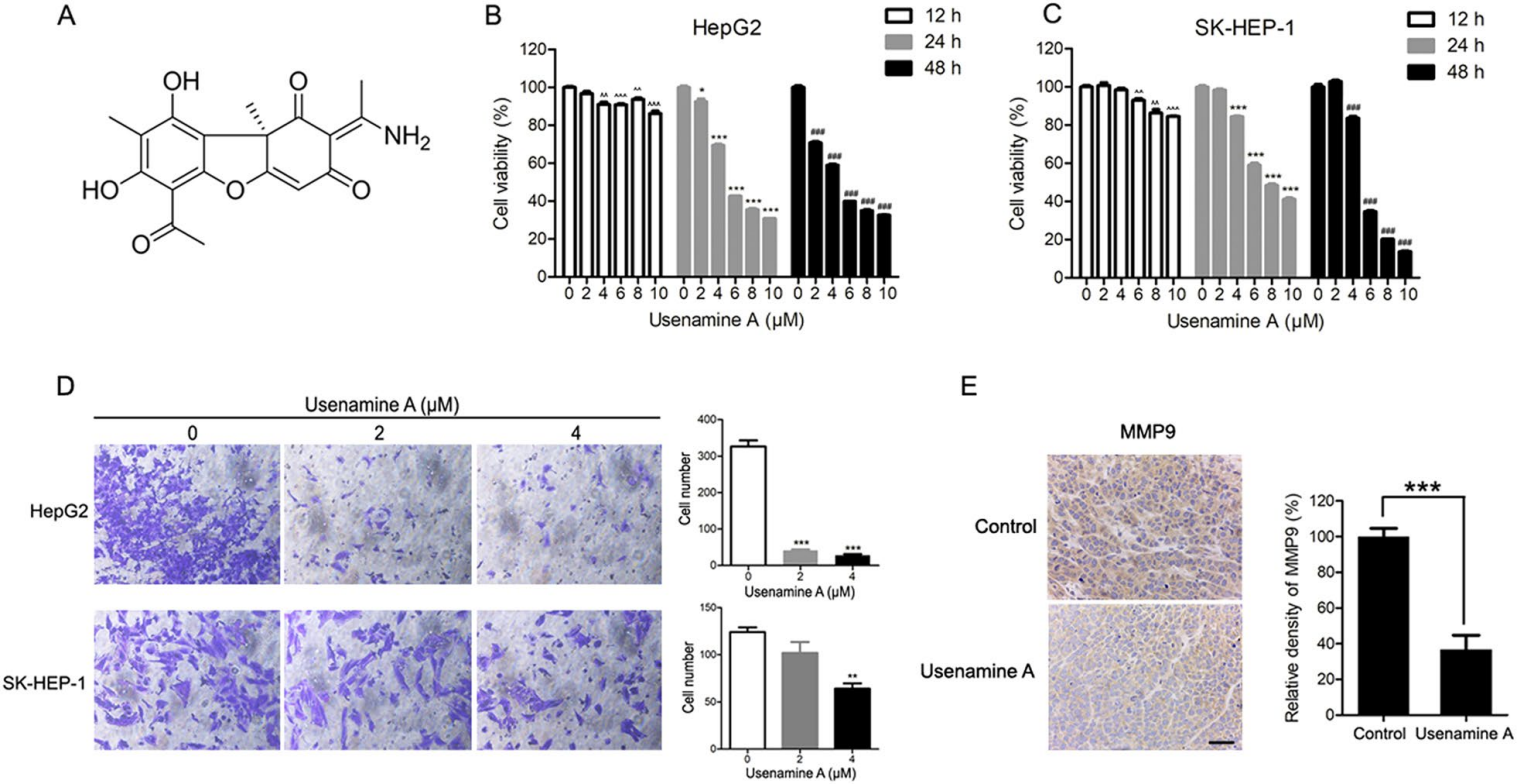
Usenamine A inhibited the proliferative and invasive capability of human HCC cells. **A** Structural formula of usenamine A. **B**, **C** Usenamine A (0/2/4/6/8/10 µM) was added to treat HepG2 (**B**) and SK-HEP-1 (**C**) cells for 12/24/48 h, respectively. Cell viability was examined by a CCK-8 assay. **D** Usenamine A was added to treat both cell lines for the Transwell assay. Left panel, typical images (200 ×); Right panel, quantification analysis. **E** Immunohistochemical analysis of MMP9 expression in tumor tissues from the HepG2 tumor-bearing nude mice with or without usenamine A treatment. Scale bar = 50 µm. Image-Pro Plus software was used for quantification. ^^^^*P* < 0.01, ^^^^^*P* < 0.001, ^*^P < 0.05, ^**^P < 0.01, ^***^P < 0.001, ^#^*P* < 0.05, ^##^*P* < 0.01, and ^###^*P* < 0.001. The data are representative of three independent experiments, and each was performed at least in triplicate

Usenamine A, a novel benzofuran derivative isolated from the medicinal lichen *Usnea longissima* in our laboratory (Fig. 1A) [10], has previously demonstrated potent effects against hepatoma [11]. Usenamine A inhibited the growth of human HCC cells by targeting Myosin-9 [11]. Additionally, usenamine A suppressed UBA5 expression and upregulated autophagy in breast cancer cells [12]. Nonetheless, the mechanisms of the anti-HCC effect of usenamine A still need further clarification.

According to our findings, usenamine A treatment suppressed the proliferation and invasion of human HCC cells, and inhibited ID1 expression by suppressing AKT/mTOR/STAT3 signaling and promoting ubiquitin–proteasome-mediated degradation in HCC cells. Usenamine A represents a potential therapeutic option for treating HCC cases characterized by aberrant AKT/mTOR/STAT3/ID1 signaling.

## Methods

### Reagents, antibodies, and drug

We obtained Dulbecco’s Modified Eagle’s Medium (DMEM, 10–013-CV), fetal bovine serum (FBS, 35–010-CV), 0.25% trypsin–EDTA (25–053-CI), and penicillin–streptomycin antibiotics (30–002-CI) from Corning Life Sciences (Steuben County, New York, USA); Matrigel was acquired from BD Biosciences (San Jose, CA, USA, 356234); Cell Counting Kit-8 was obtained from Dojindo (Kumamoto, Japan, CK04); anti-β-actin (sc-47778) and anti-ID1 (sc-133104) antibodies from Santa Cruz Biotechnology (Santa Cruz, CA, USA); anti-AKT (4691), anti-p-AKT (Thr473, 4060), anti-mTOR (2983), anti-p-mTOR (S2448, 5536), anti-p-STAT3 (Y-705, 9145), anti-STAT3 (4904), anti-S6 (2317), anti-p-S6 (Ser235/236, 2211), and anti-ubiquitin (3933) antibodies from Cell Signaling Technology (Danvers, MA, USA); and anti-mouse (sc-516102) and anti-rabbit (sc-2357) IgG-HRP antibodies from Santa Cruz Biotechnology (Santa Cruz, CA, USA). Bis–Tris NuPAGE gels (4–12%) were purchased from Invitrogen (Carlsbad, CA, USA), and the enhanced chemiluminescence (ECL) detection kit was obtained from Applygen Technologies Inc. (Beijing, China). Usenamine A (Purity > 99.8%) was obtained as described previously [11], and prepared as 20 and 40 mM stock solutions through dissolution into DMSO before subsequent dilutions for further analysis.

### Cell culture

Human HCC cell lines HepG2 and SK-HEP-1 were provided by American Type Culture Collection (Manassas, VA, USA) and cultivated in DMEM complete medium (containing 10% FBS, 100 U mL^–1^ penicillin, as well as 100 µg mL^–1^ streptomycin). The cells were cultured at 37 °C in a 5% CO_2_ humidified atmosphere.

### Cell proliferation assay

After cell inoculation (3.5 × 10^4^–4.0 × 10^4^ cells/mL) into 96-well plates, the culture medium was replaced with a freshly prepared complete medium containing various doses of usenamine A. After 12-, 24- and 48 h of usenamine A treatment, 10 µL of CCK-8 solution was added to each well. Following a 2 h incubation at 37 °C, optical density measurements were performed at 450 nm using a microplate reader (Perkin-Elmer, Waltham, MA, USA).

### Cell invasion assay

HepG2 and SK-HEP-1 cells were cultured for 12 h in the serum-free medium to reduce serum interference. The Transwell system was established as previously described [13]. After collection, the cells were resuspended in the serum-free medium containing varying doses of usenamin A. The cells resuspended in the drug-containing serum-free medium (200 µL) were introduced into the top chamber, while the complete medium with 10% FBS (750 µL) was introduced into the bottom chamber. After incubation for different periods, a cotton swab was used to remove cells from the upper chamber surface. The cells on the lower surface of the chamber were then fixed by adding ethanol and incubating for a 15 min period, followed by 10 min staining using crystal violet. The chamber was washed twice with PBS. The number of invasive cells in five random fields was counted, and photographs were taken using an inverted microscope.

### RNA interference

GenePharma (Shanghai, China) carried out siRNA synthesis. By using Lipofectamine 2000, siRNAs were transfected into cells seeded within the 6-well plates as per specified protocols. The siRNA sequences used are as follows:

STAT3: 5′-UCCAGUUUCUUAAUUUGUUGACGGGUC-3′;

ID1: 5′-CGACAUGAACGGCUGUUACTT-3′;

Negative Control (NC): 5′-UUCUCCGAACGUGUCACGUTT -3′.

### Quantitative real-time PCR

Total RNA extraction of human HCC cells was carried out using the E.Z.N.A.^®^ Total RNA Kit I (Omega Bio-Tek, Norcross, GA, USA). The extracted RNA was then reverse-transcribed into cDNA using the PrimeScript RT Reagent Kit (TaKaRa, Dalian, China) according to the manufacturer’s instructions. The cDNA (4 µL) was then used as a template in quantitative PCR with TransStart Top Green qPCR SuperMix (TransGen Biotech, Beijing, China). Sequences of all primers utilized are as follows:

ID1 forward: 5′-CTGCTCTACGACATGAACGG-3′, ID1 reverse: 5′-GAAGGTCCCTGATGTAGTCGAT-3′; and β-actin forward: 5′-TCACCCACACTGTGCCCATCTAC-3′, β-actin reverse: 5′-GAGTACTTGCGCTCAGGAGGAGC-3′.

### Expression profiling

RNA isolated in HepG2 cells with or without usenamine A treatment were subjected to microarray analysis using the Affymetrix GeneChip Human Transcriptome Array (HTA) 2.0 on the Affymetrix GeneChip 645 System. Affymetrix Expression console software was used for data analysis by Shanghai Biotechnology Corp. (Shanghai, China).

### Immunoblotting

PBS was added to wash cells treated with usenamine A twice before cell harvesting using the lysis buffer (60 mM Tris, pH 6.8, 0.1 M DTT, 10% glycerol, 2% SDS). Immunoblotting was carried out for protein detection as previously described [14].

### Immunoprecipitation

MG-132 (20 µM) was added to treat HepG2 and SK-HEP-1 cells, followed by 8 h of 8 µM usenamine A treatment. After collecting total cell lysates, an anti-ID1 antibody (3 µg) was added and incubated for 18 h at 4 °C. This was followed by a 2 h incubation using Protein A/G Sepharose beads at 4 °C. We subsequently rinsed immune complexes four times using a washing buffer before immunoblotting using an anti-ubiquitin antibody.

### Chromatin immunoprecipitation (ChIP) assay

The ChIP assay was used for analyzing DNA–protein interactions using an anti-STAT3 antibody and the ChIP assay kit (Millipore, catalog number: 17–295) according to provided instructions. PCR assay was performed with purified DNA, using the following primer sequences: the putative STAT3 binding site region of human ID1, for primer 1, 5′-TGCGACCCGCCTGAAT-3′ (forward), and 5′-CTGCGAGTCTCCCAAC-3′ (reverse); for primer 2, 5′-AATGGAGCTGGAGAAGTG-3′ (forward), and 5′-TGGAAGGAGGCAAGGA-3′ (reverse).

### Immunohistochemistry (IHC) analysis

Tumor samples were collected from the HepG2 tumor-bearing nude mice with or without usenamine A treatment, as described previously [11], followed by fixation using 4% paraformaldehyde and paraffin embedding. Antibodies against MMP-9, ID1, and p-STAT3 were used for the immunohistochemistry analysis according to a previous description [15].

### Statistical analysis

Results are represented as mean ± SEM. A two-tailed Student’s *t*-test was utilized to evaluate the significant difference between two groups using GraphPad Prism 5.0. *P* < 0.05 signifies statistical significance.

## Results

### Usenamine A suppresses human hepatocellular carcinoma cell proliferation and invasion

A CCK-8 assay was performed to determine the effects of usenamine A on the proliferation of human HCC cell lines (HepG2 and SK-HEP-1). Usenamine A significantly suppressed the proliferation of the above mentioned cell lines in a dose- and time-dependent manner (Fig. 1B, C). For usenamine A, its IC_50_ values in these two human HCC cell lines for a 48-h period were < 10 µM. Transwell assay was used to examine the invasive capacity of human HCC cells treated with usenamine A in vitro. Both the cell lines were exposed to usenamine A treatment at specific doses for different time intervals. Subsequently, following usenamine A treatment, a decrease in cell number was observed across the Matrigel-coated membrane (Fig. 1D). Furthermore, IHC analysis of tumor samples obtained from the HepG2 tumor-bearing nude mice revealed a reduction in MMP-9 expression upon usenamine A treatment, suggesting the inhibitory effect of usenamine A on the metastatic potential of the tumors in mice (Fig. 1E).

### Usenamine A inhibits ID1 expression within human HCC cells

**Fig. 2:**
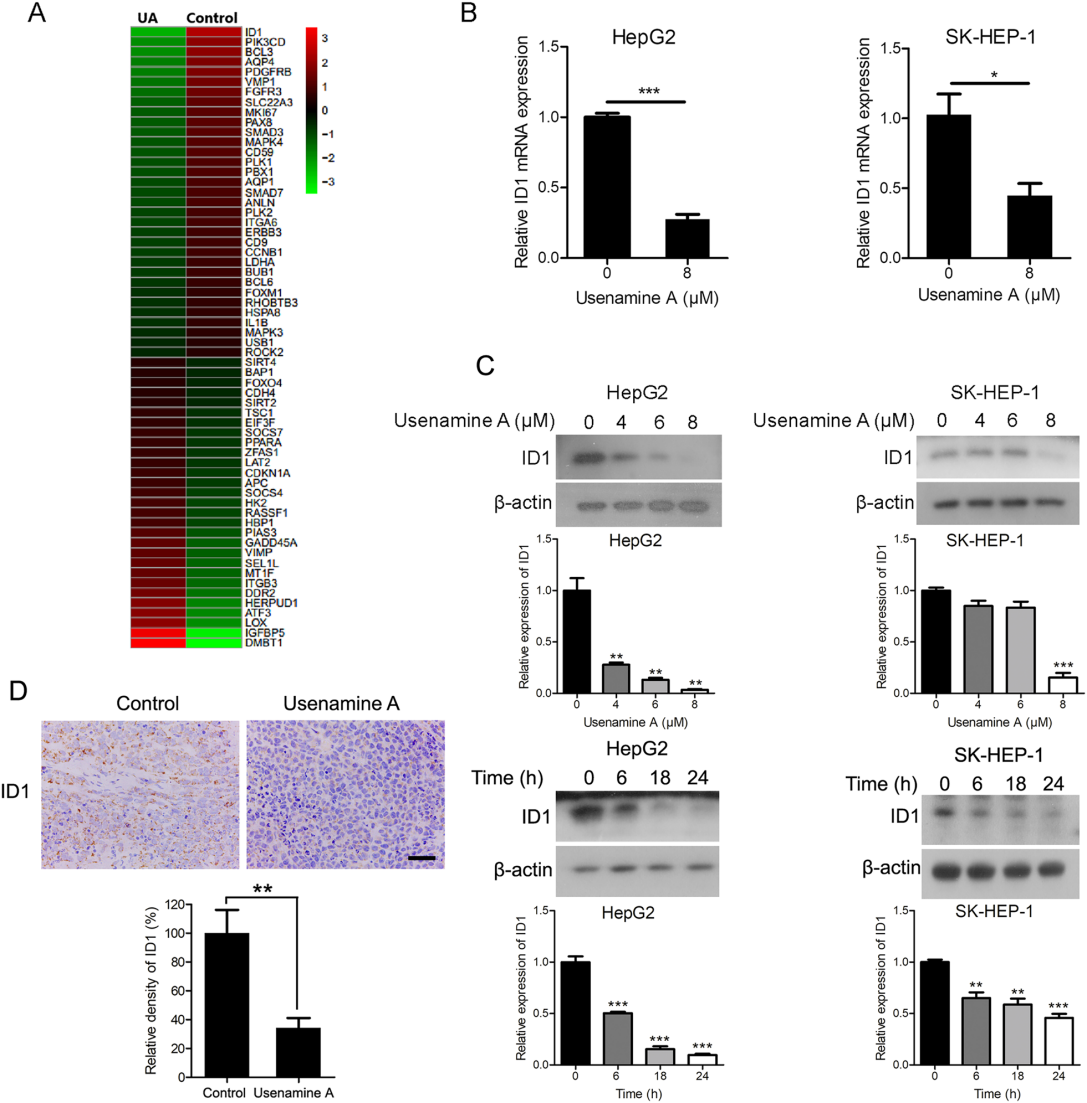
Usenamine A suppressed ID1 expression in human HCC cells. **A** Hierarchical clustering analysis on cancer-associated genes with differential expression in HepG2 cells with or without 24 h of usenamine A (8 µM) treatment. Red and green indicate high and low expression, respectively. **B** Total RNAs were collected in either cell lines with or without 24 h of usenamine A (8 µM) treatment and used for quantitative real-time PCR. **C** Cell lysates from both cell lines were prepared after 24 h of usenamine A treatment (0/4/6/8 µM) or 8 µM usenamine A for specific durations (0/6/18/24 h), and subsequently used for immunoblotting. **D** Immunohistochemical analysis of ID1 expression in tumor tissues from the HepG2 tumor-bearing nude mice with or without usenamine A treatment. Scale bar = 50 µm. Image-Pro Plus software was used for quantification. ^*^P < 0.05, ^**^P < 0.01, and ^***^P < 0.001. The data are representative of three independent experiments, and each was performed at least in triplicate

Gene expression profiling suggested that ID1 level was reduced in HepG2 cells upon usenamine A treatment (Fig. 2A). As verified by quantitative real-time PCR assay, usenamine A treatment significantly reduced the ID1 mRNA levels in HepG2 and SK-HEP-1 cells (Fig. 2B). Additionally, ID1 protein level was decreased after usenamine A treatment in both HCC cell lines in a dose- and time-dependent manner (Fig. 2C). Furthermore, ID1 expression was decreased in the mice tumor tissues subjected to usenamine A treatment compared to that of the controls (Fig. 2D). In summary, usenamine A treatment suppressed ID1 expression in human HCC cells.

### ID1 was implicated in the inhibition of usenamine A on human HCC cell proliferation and invasion

**Fig. 3:**
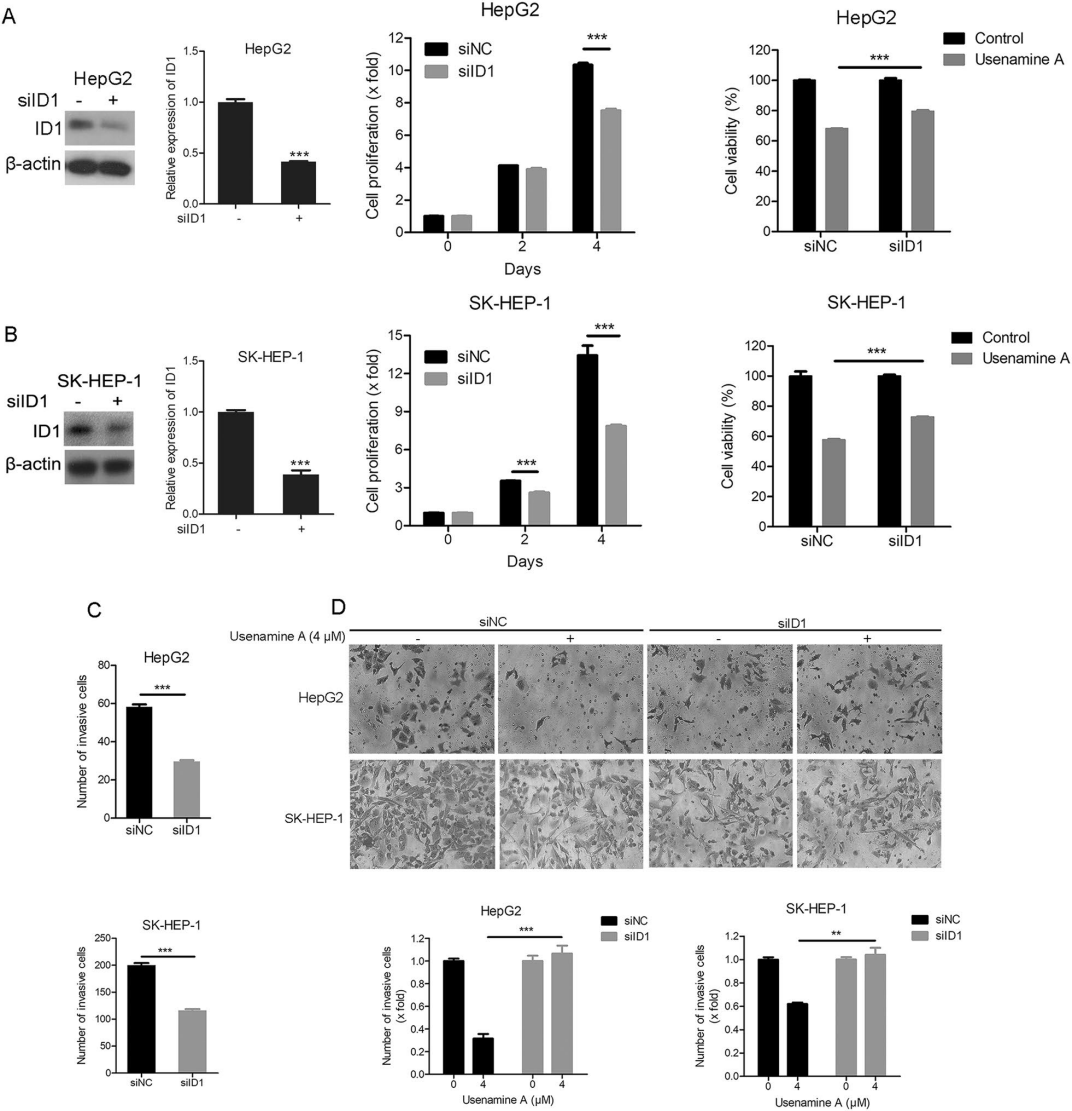
ID1 participated in the inhibitory effects of usenamine A against human HCC cell proliferation and invasion. **A**, **B** Total cell lysates were prepared from HepG2 (**A**) or SK-HEP-1 (**B**) cells subjected to ID1 or negative control (NC) siRNAs transfection and used for immunoblotting. Either cell line subjected to ID1 or NC siRNAs transfection was used for cell proliferation assay. Usenamine A (4 µM) was added for 48-h treatment of either cell line subjected to ID1 or NC siRNAs transfection. Cell viability was examined using CCK-8 assay. **C**, **D** Either cell line subjected to ID1 or NC siRNAs transfection was used for Transwell assay (**C**), and Transwell assay with or without usenamine A (4 µM) treatment (**D**). Upper panel, typical images (200 ×); Lower panel, quantification analysis. ^**^P < 0.01 and ^***^P < 0.001. The data are representative of three independent experiments, and each was performed at least in triplicate

We further investigated whether ID1 was involved in the antiproliferative and antimetastatic functions of usenamine A in HCC cells. ID1 expression was silenced through RNA interference in HepG2 cells, resulting in the suppression of HepG2 cell growth (Fig. 3A). Additionally, ID1 knockdown suppressed the inhibitory function of usenamine A on HepG2 cell growth (Fig. 3A). According to Fig. 3B, similar findings were observed in SK-HEP-1 cells. Additionally, ID1 knockdown restrained HepG2 and SK-HEP-1 cell invasion (Fig. 3C). Thus, ID1 positively regulated human HCC cell proliferation and invasion. Intriguingly, ID1 depletion impaired usenamine A’s inhibition against the invasion of the above two cell lines (Fig. 3D). Therefore, the downregulation of ID1 contributes to the inhibitory functions of usenamine A on HCC cell proliferation and invasion.

### Usenamine A inhibited STAT3 activity in human HCC cells

**Fig. 4:**
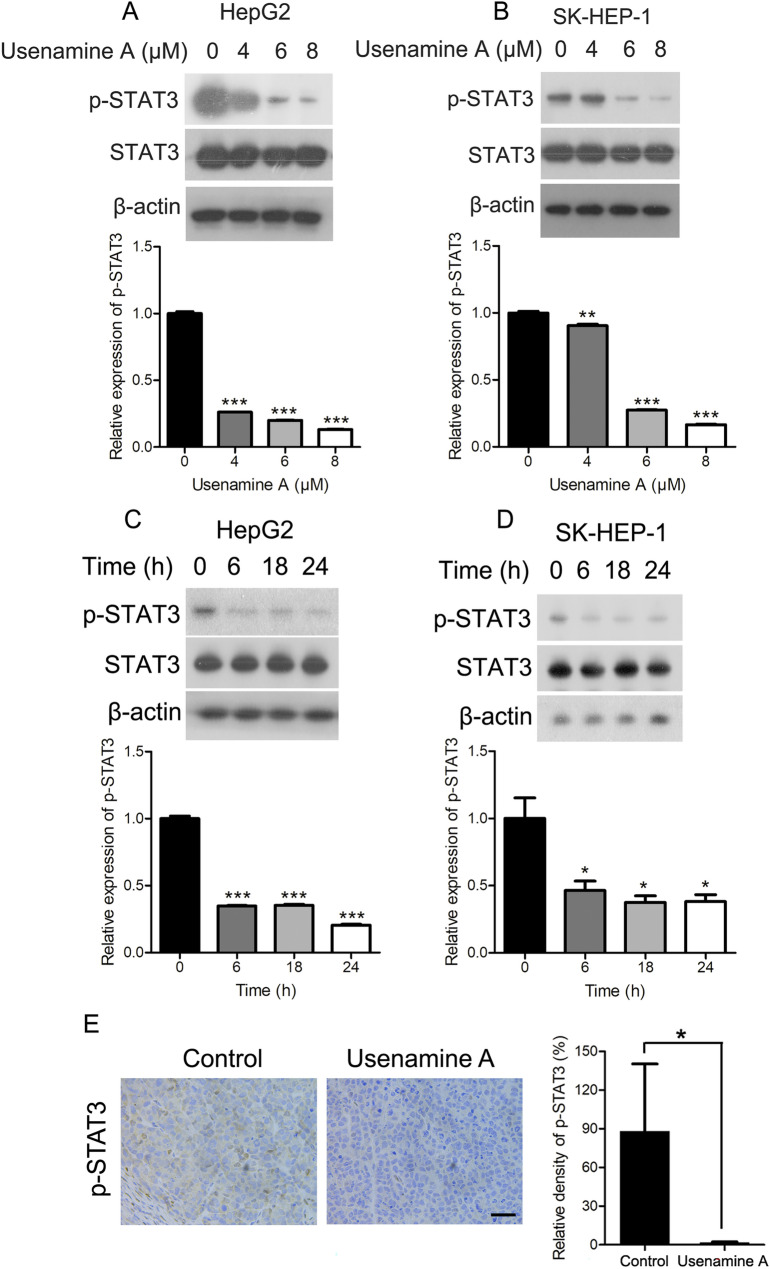
Usenamine A inhibited STAT3 activity in human HCC cells. **A**, **B** Total cell lysates were prepared based on HepG2 (**A**) and SK-HEP-1 (**B**) cells subjected to 24 h treatment using 0/4/6/8 µM usenamine A and used for immunoblotting. **C**, **D** Total cell lysates were prepared in the cell lines after 8 µM usenamine A treatment for specified durations (0/6/18/24 h) and used for immunoblotting. **E** Immunohistochemical analysis of p-STAT3 protein levels in tumor tissues from the HepG2 tumor-bearing nude mice with or without usenamine A treatment. Scale bar = 50 µm. Image-Pro Plus software was used for quantification analysis. ^*^P < 0.05, ^**^P < 0.01, ^***^P < 0.001. The data are representative of three independent experiments, and each was performed at least in triplicate

STAT3 is known to be hyperactivated in various cancer types, such as liver cancer [16]. This hyperactivation is closely associated with aberrant tumor cell proliferation and increased metastasis [5, 16]. As revealed by western blotting, STAT3 phosphorylation was substantially decreased after usenamine A treatment at different concentrations in both HepG2 and SK-HEP-1 cells (Fig. 4A, B). Moreover, 8 µM usenamine A was added to treat both cell lines at different durations and the level of phosphorylated STAT3 was also markedly reduced (Fig. 4C, D). Additionally, the IHC assay suggested a significant reduction in p-STAT3 protein levels in the nude mice tumor tissues receiving usenamine A treatment (Fig. 4E). Therefore, usenamine A suppressed STAT3 activity in human HCC cells.

### STAT3 participated in the function of usenamine A in suppressing HCC cell proliferation and invasion

**Fig. 5:**
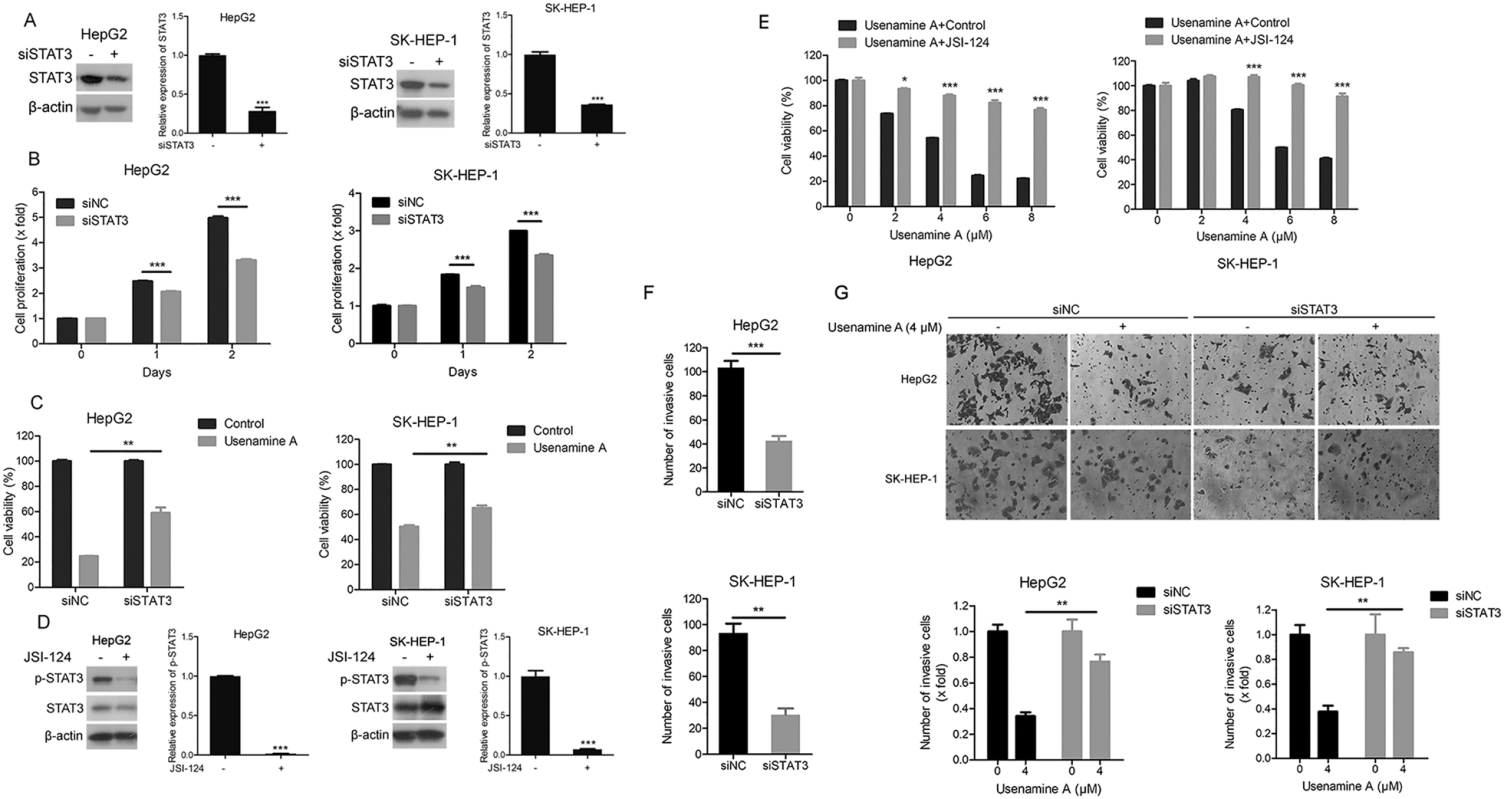
STAT3 was required for inhibiting human HCC cell proliferation and invasion after treatment using usenamine A. **A** Total cell lysates were prepared from HepG2 or SK-HEP-1 cells subjected to STAT3 or negative control (NC) siRNAs transfection and used for immunoblotting. **B** Cell proliferation analysis was performed in both cell lines transfected with STAT3 or NC siRNAs. **C** After STAT3 or NC siRNAs transfection, both cell lines were treated with or without 4 µM usenamine A for 48-h. Cell viability was examined using a CCK-8 assay. **D** Total cell lysates were prepared from HepG2 or SK-HEP-1 cells subjected to 24-h treatment with or without JSI-124 (10 µM) and used for immunoblotting. **E** HepG2 or SK-HEP-1 cells were subjected to 48 h of usenamine A treatment (0/2/4/6/8 µM) with or without JSI-124 (10 µM) treatment. Cell viability was examined using a CCK-8 assay. **F**, **G** Either cell line subjected to STAT3 or NC siRNAs transfection was used for Transwell assay (**F**), and Transwell assay with or without usenamine A (4 µM) treatment (**G**). Upper panel, typical images (200 ×); Lower panel, quantification analysis. ^*^P < 0.05, ^**^P < 0.01, and ^***^P < 0.001. The data are representative of three independent experiments, and each was performed at least in triplicate

First, STAT3 expression was silenced through RNA interference in both HCC cell lines (Fig. 5A). According to Fig. 5B, STAT3 depletion dramatically suppressed the proliferation of both cell lines. Moreover, STAT3 silencing suppressed the function of usenamine A in inhibiting HCC cell proliferation (Fig. 5C). Similarly, the use of JSI-124, an inhibitor of STAT3, significantly reduced the impact of usenamine A on both cell lines (Fig. 5D, E). Consequently, inhibition of STAT3 activity contributed to the inhibitory effects of usenamine A on HCC cell proliferation. Moreover, the suppression of invasion in both HCC cell lines was observed upon silencing of STAT3 (Fig. 5F). Furthermore, siRNA-mediated silencing of STAT3 weakened the inhibitory effect of usenamine A on HCC cell invasion (Fig. 5G). These findings suggest that STAT3 plays a crucial role in the function of usenamine A in inhibiting HCC cell proliferation and invasion.

### STAT3 positively regulated ID1 expression at the transcriptional level

**Fig. 6:**
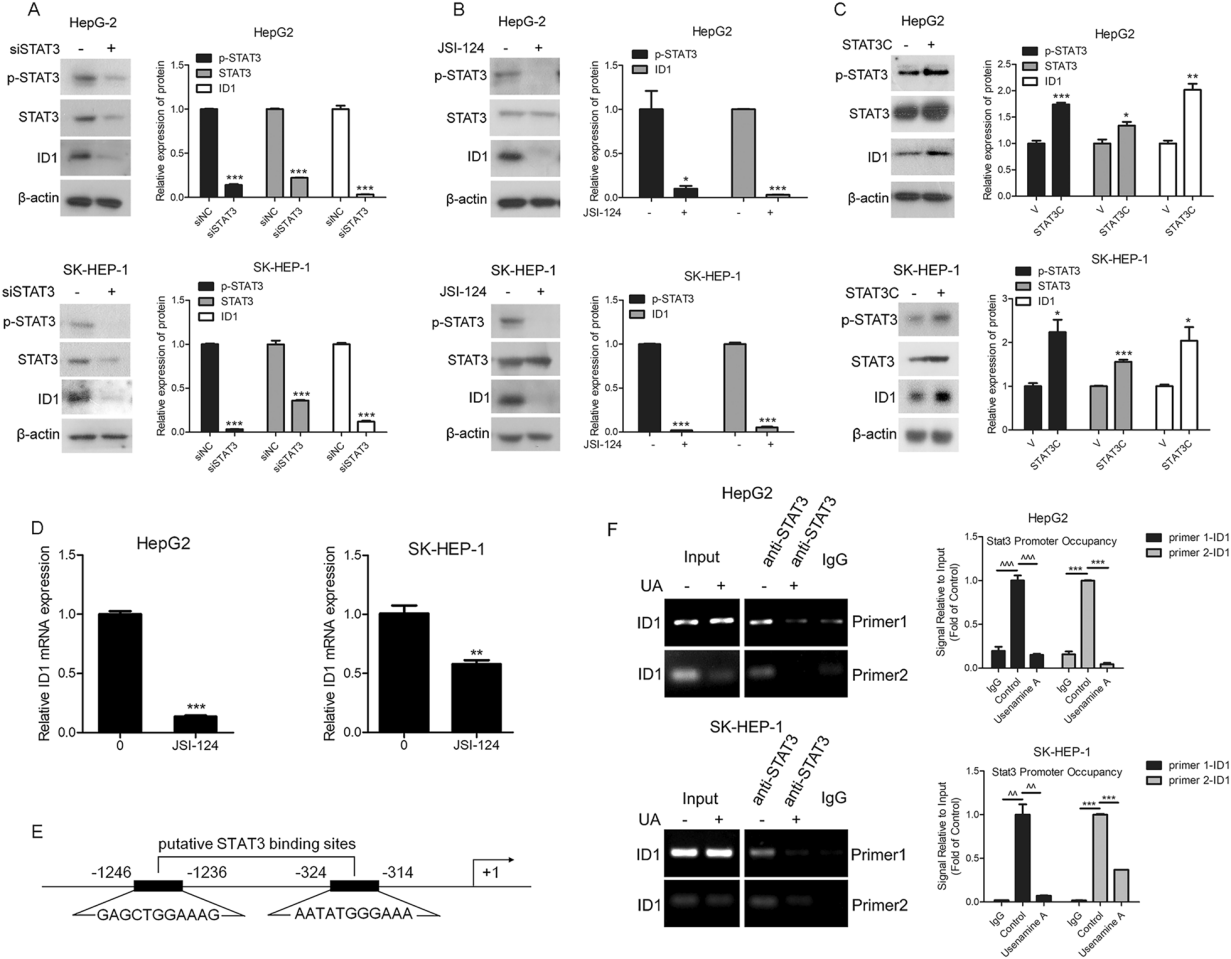
STAT3 positively regulated ID1 expression at the transcriptional level. **A** Total cell lysates were prepared from HepG2 or SK-HEP-1 cells subjected to STAT3 or NC siRNAs transfection and used for immunoblotting. **B** Total cell lysates were prepared from HepG2 or SK-HEP-1 cells subjected to 24 h of JSI-124 (10 µM) treatment and used for immunoblotting. **C** Total cell lysates were prepared from HepG2 or SK-HEP-1 cells subjected to constitutively activated STAT3 (STAT3C) transfection and used for immunoblotting. **D** Total RNAs were isolated from both cell lines with or without JSI-124 (10 µM) treatment for 24 h and utilized for quantitative real-time PCR. **E** Sketch map showing the putative STAT3-binding sequences in the ID1 gene promoter. **F** Both cell lines, subjected to 24 h treatment with or without 8 µM usenamine A, were used for chromatin immunoprecipitation assay using the anti-STAT3 antibody. The normal rabbit IgG antibody served as the negative control. PCR amplification was conducted with primers around the putative STAT3-binding sites. ^*^P < 0.05, ^**^P < 0.01, ^***^P < 0.001, ^^^^*P* < 0.01, and ^^^^^*P* < 0.001. The data are representative of three independent experiments, and each was performed at least in triplicate

The knockdown of STAT3 by RNA interference decreased the ID1 protein levels in both HCC cell lines (Fig. 6A). Moreover, the inhibition of STAT3 activity with JSI-124 (the specific STAT3 inhibitor) reduced ID1 protein levels in both HCC cell lines (Fig. 6B). Furthermore, the overexpression of constitutively activated STAT3 (STAT3C) in HepG2 and SK-HEP-1 cells resulted in increased ID1 protein levels (Fig. 6C). Quantitative real-time PCR analysis unveiled that the inhibition of STAT3 reduced the levels of ID1 mRNA in both HCC cell lines (Fig. 6D). These findings collectively indicate that STAT3 plays a positive regulatory role in ID1 expression. To determine whether STAT3 directly regulates ID1 expression, we examined the consensus binding sites of STAT3 in the promoter region of the ID gene. Two potential STAT3 binding sequences (− 1246/− 1236 GAGCTGGAAAG and − 324/− 314 AATATGGGAAA) were identified within the ID1 gene promoter (Fig. 6E). Subsequently, ChIP assay results demonstrated that STAT3 bound to the putative binding sites in the ID1 gene promoter region in both HCC cell lines. However, treatment with usenamine A inhibited STAT3 binding to these candidate binding sites in the ID1 promoter region (Fig. 6F), indicating that usenamine A treatment prevented STAT3 from binding to the ID1 promoter region. Consequently, the downregulation of ID1 expression observed in HCC cells exposed to usenamine A can be attributed to the inhibition of STAT3-mediated ID1 gene transcription.

### Usenamine A inhibited AKT/mTOR/STAT3/ID1 signaling in human HCC cells

**Fig. 7:**
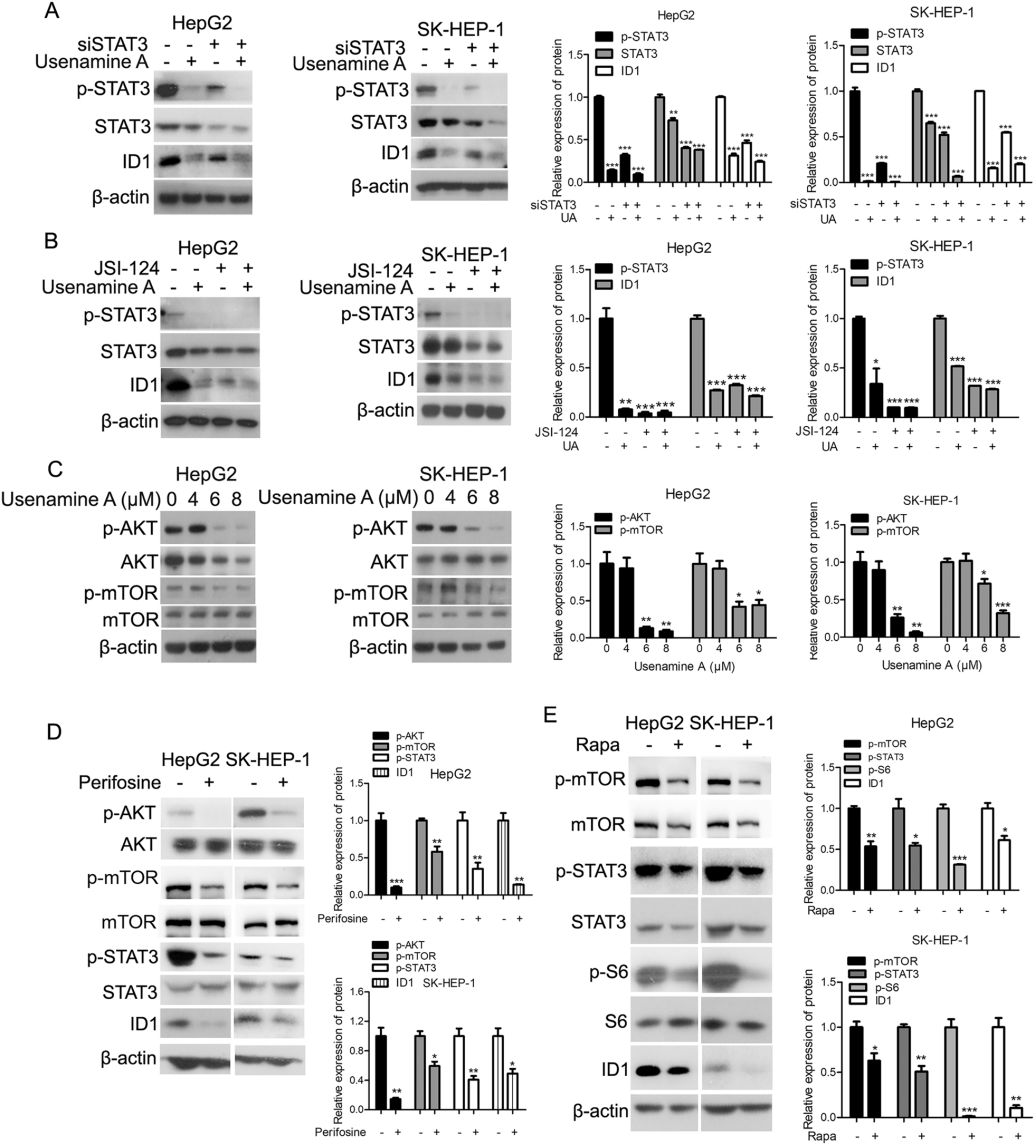
Usenamine A inhibited the AKT/mTOR/STAT3/ID1 signaling axis in human HCC cells. **A** Total cell lysates were prepared from both HCC cell lines subjected to STAT3 or NC siRNAs transfection in the absence or presence of 8 µM usenamine A and used in immunoblotting. **B** Total cell lysates were prepared from both HCC cell lines subjected to treatment with or without 10 µM JSI-124 in the absence or presence of 8 μM usenamine A and used for immunoblotting. **C** Total cell lysates were prepared from both HCC cell lines subjected to 24 h usenamine A treatment (0/4/6/8 µM) and used for immunoblotting. **D** Total cell lysates were prepared from both HCC cell lines subjected to 24 h treatment with or without perifosine (10 µM) and used for immunoblotting. **E** Total cell lysates were prepared from both HCC cell lines subjected to 24 h treatment with or without rapamycin (10 nM) and used for immunoblotting. ^*^P < 0.05, ^**^P < 0.01, and ^***^P < 0.001. The data are representative of three independent experiments, and each was performed at least in triplicate

STAT3 activity was inhibited by transfecting siRNAs or adding specific inhibitors to both HCC cell lines to determine if STAT3 was related to the impact of usenamine A on ID1 expression. The results presented in Fig. 7A demonstrated that siRNA-mediated genetic deficiency of STAT3 compromised the suppressive impact of usenamine A on ID1 expression. Additionally, the pharmacological inhibition of STAT3 using JSI-124 attenuated the usenamine A-induced suppression of ID1 expression (Fig. 7B). Taken together, these findings indicated the essential role of STAT3 in the usenamine A-mediated inhibition of ID1 expression. Previous studies have identified STAT3 as a downstream target of the AKT/mTOR pathway [17]. After exposure to usenamine A, mTOR and AKT phosphorylation levels were decreased, suggesting that usenamine A inhibited AKT/mTOR pathway in both HCC cell lines (Fig. 7C). Moreover, the inhibition of AKT with perifosine (an AKT inhibitor) [18] downregulated STAT3 activity and ID1 levels in both HCC cell lines (Fig. 7D). Rapamycin is a specific mTOR inhibitor, and as an important downstream effector of mTOR, the inhibition of ribosomal protein S6 activity is considered to be a marker of decreased mTOR activity [19]. As shown in Fig. 7E, treatment using rapamycin effectively inhibited the activity of mTOR and S6, and inhibition of mTOR suppressed STAT3 phosphorylation and ID1 expression in both HCC cell lines, suggesting that STAT3/ID1 axis was controlled by the AKT/mTOR signaling pathway in both HCC cell lines. Overall, usenamine A inhibited AKT/mTOR/STAT3/ID1 signaling pathway in human HCC cells.

### Usenamine A promoted ID1 protein degradation through the ubiquitin–proteasome pathway

**Fig. 8:**
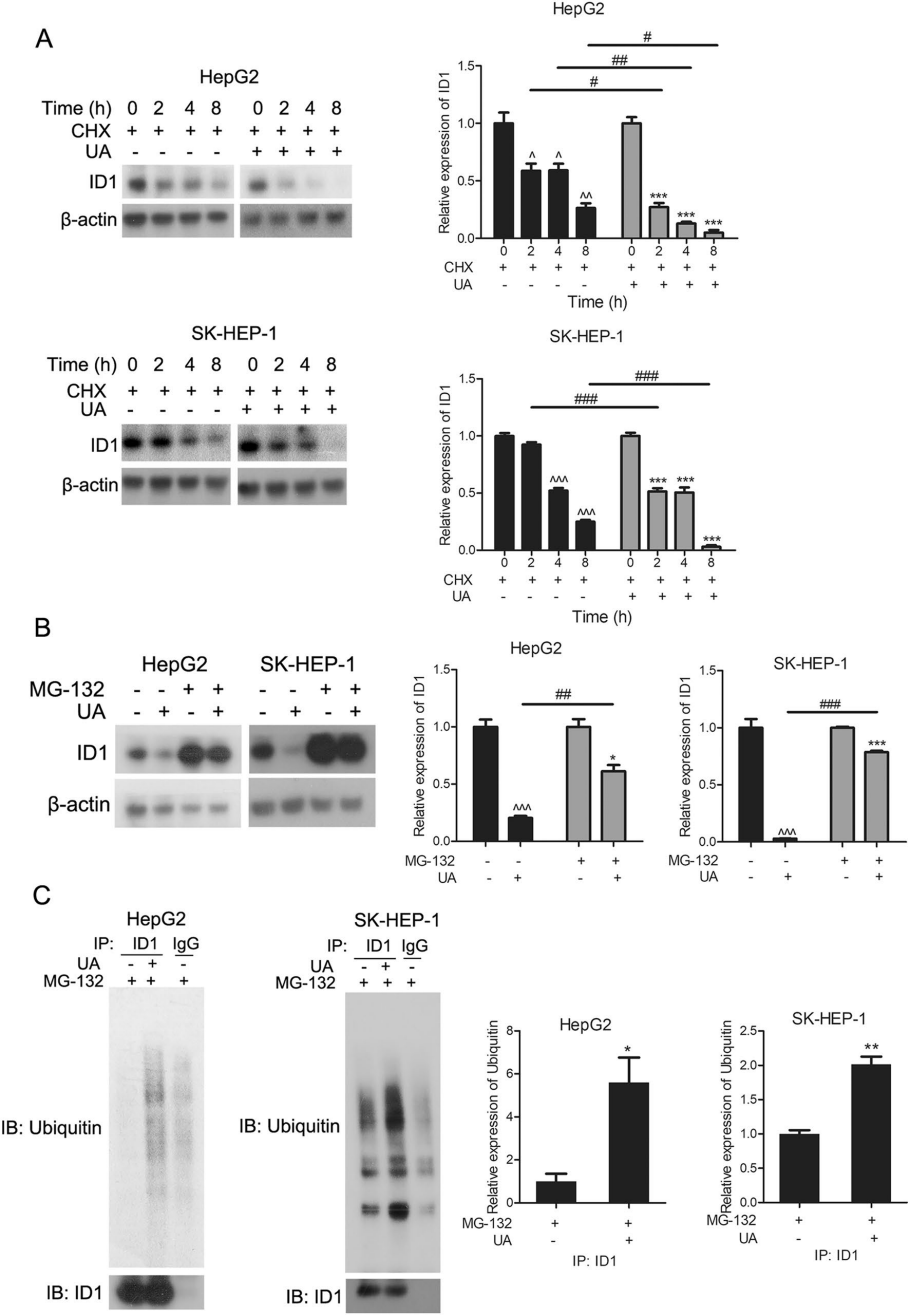
Usenamine A promoted ubiquitin–proteasome-dependent degradation of ID1. **A** Total cell lysates were prepared from both cell lines subjected to CHX (50 µM) treatment for specified durations (0/2/4/8 h) with or without usenamine A (8 µM) treatment and used for immunoblotting. **B** Total cell lysates were prepared from both cell lines subjected to 6 h treatment with or without MG132 (20 µM) in the absence or presence of usenamine A (8 µM) for immunoblotting. **C** Total cell lysates were prepared from both cell lines subjected to treatment with or without usenamine A (8 µM) in the presence of MG132 (20 μM) for 8 h and used for immunoprecipitation with an anti-ID1 antibody. ^^^*P* < 0.05, ^^^^*P* < 0.01, ^^^^^*P* < 0.001, ^*^P < 0.05, ^**^P < 0.01, ^***^P < 0.001, ^#^*P* < 0.05, ^##^*P* < 0.01, and ^###^*P* < 0.001. The data are representative of three independent experiments, and each was performed at least in triplicate

ID1 expression can be controlled via a ubiquitin-dependent proteolytic pathway [20, 21]. In this study, we investigated the potential involvement of ubiquitin-dependent degradation in the reduced expression of ID1 in human HCC cells following treatment with usenamine A. As depicted in Fig. 8A, ID1 protein level decreased in both HCC cell lines exposed to cycloheximide (the protein synthesis inhibitor) [22] in a time-dependent manner, and usenamine A treatment promoted the downregulation of ID1 protein levels in the presence of cycloheximide, indicating that usenamine A treatment enhanced the degradation of ID1 protein in HCC cell lines. Furthermore, the inhibitory effect of usenamine A on ID1 protein levels was attenuated when treated with the specific proteasome inhibitor MG-132 [23] in both HCC cell lines (Fig. 8B). Moreover, an immunoprecipitation assay demonstrated that usenamine A increased the abundance of ubiquitinated ID1 protein in the presence of MG-132 in both HCC cell lines (Fig. 8C). These findings collectively suggest that usenamine A partially reduces the ID1 protein level by promoting its degradation through the ubiquitin-mediated pathway.

## Discussion

Naturally-derived products, such as curcumin, berberine, and resveratrol, exert good clinical effects as cancer therapies [24, 25]. Usenamine A, a new benzofuran derivative first isolated from the medicinal lichen *Usnea longissima* in our laboratory [10], is known to be potent against hepatoma [11]. In this study, usenamine A exhibited antihepatoma activity partially by inhibiting ID1 expression through the downregulation of the AKT/mTOR/STAT3 signaling pathway and the induction of ubiquitin–proteasome-mediated ID1 degradation.

ID1 expression is markedly upregulated in diverse human tumors, such as HCC [9]. Elevated ID1 expression is strongly associated with an increased risk of HCC, and ID1 is the marker used to predict the occurrence of HCC [26]. Studies have shown that ID1 promotes human HCC cell proliferation by suppressing the p16^INK4a^/pRB signaling pathway [27], and activation of the androgen receptor (AR) enhances human HCC cell migration and invasion by upregulating ID1 expression [28]. Based on the microarray data, we observed a decrease in both ID1 mRNA and protein levels in human HCC cells upon treatment with usenamine A. Furthermore, as ID1 is known to undergo degradation through the ubiquitin–proteasome pathway, we investigated whether usenamine A affected ID1 protein degradation. Interestingly, our findings revealed that usenamine A treatment facilitated the degradation of ID1 protein via the ubiquitin–proteasome pathway. Moreover, we identified that ID1 plays a positive role in promoting the proliferative and metastatic capabilities of human HCC cells, and the depletion of ID1 attenuated the ability of usenamine A to suppress HCC cell proliferation and invasion. Consequently, the down-regulation of ID1 contributed to the antihepatoma effect of usenamine A.

STAT3, a key transcription factor, is activated by phosphorylation. Phosphorylated STAT3 undergoes dimerization and translocates into the cell nucleus, where it binds to target genes and promotes their transcription [16]. STAT3 is hyperactivated in different tumors, such as liver cancer [29, 30]. The examination of pathological tissue samples obtained from liver cancer patients revealed a correlation between higher STAT3 activity levels and larger tumor volume, poorer prognosis, and increased postoperative recurrence rate [31, 32]. STAT3 is frequently overactivated in liver cancer. Hence, inhibiting STAT3 is a possible anti-liver cancer therapeutic strategy. In human colon cancer cells, ectopic STAT3 expression increased ID1 mRNA and protein expression [33]. STAT3 inhibition decreased the upregulation of ID1 levels caused by heparin-binding epidermal growth factor (HB-EGF) in neuroblastoma cells [34]. To gain a deeper insight into the mechanisms underlying the reduction of ID1 by usenamine A treatment, we hypothesized the existence of a STAT3/ID1 axis in HCC cells. Our findings demonstrate that usenamine A effectively inhibits STAT3 activity in HCC cells. Additionally, the down-regulation of STAT3 plays a crucial role in usenamine A’s ability to suppress HCC cell proliferation and invasion. Furthermore, we observed that STAT3 positively regulates both the mRNA and protein expression of ID1 in human HCC cells. Through our analysis, we identified two putative binding sites (− 1246/− 1236 GAGCTGGAAAG and − 324/− 314 AATATGGGAAA) within the promoter region of the ID1 gene where STAT3 binds in HCC cells. This binding leads to the transcriptional upregulation of ID1 expression. Importantly, we confirmed that usenamine A inhibits ID1 expression through its modulation of STAT3. Overall, the newly identified STAT3/ID1 axis was implicated in the antihepatoma effect of usenamine A.

In cancer, the maintenance of malignant phenotypes often relies on specific key genes and pathways. Consequently, the strategic targeting of these aberrant pathways through pharmacological interventions is crucial for effective anti-tumor treatments. The AKT/mTOR signaling pathway exhibits a high dysregulation frequency in liver cancer, which has an important effect on cell proliferation and metastasis [3, 4]. AKT and mTOR are desired targets for pharmacological intervention in cancer therapy [35, 36]. Based on accumulating evidence, STAT3 is a target gene downstream of AKT/mTOR signaling in cancer cells [37–40]. In this study, usenamine A was found to be a pivotal inhibitor of the AKT/mTOR signaling pathway. Moreover, inhibition of AKT or mTOR decreased STAT3 activity and ID1 expression in human HCC cells. Therefore, usenamine A exerted an antihepatoma effect by suppressing the AKT/mTOR/STAT3/ID1 signaling cassette.

Cancer metastasis is a continuous and complicated process involving multiple steps that play critical roles in the prognosis of cancer. Roughly 90% of cancer-associated deaths can be attributed to metastasis [41]. HCC is a malignant tumor, and extrahepatic metastases are frequent in patients with HCC [42]. Hence, the attenuation of metastasis stands out as a promising therapeutic strategy in the fight against HCC [43]. Transwell assay was conducted to evaluate the metastatic capacity of HCC cells in vitro. Results reveal that usenamine A suppressed human HCC cell invasion. Moreover, IHC analysis unveiled that usenamine A treatment inhibited the metastatic potential of HCC cells. Notably, the components in the newly identified AKT/mTOR/STAT3/ID1 axis play pivotal roles in cancer metastasis [44–46]. Our study also revealed that both STAT3 and ID1 were involved in suppressing the effect of usenamine A on metastatic potential of human HCC cells. These findings highlight the potential of usenamine A as a therapeutic agent for suppressing metastasis in HCC by down-regulating the AKT/mTOR/STAT3/ID1 pathway.

## Conclusions

**Fig. 9:**
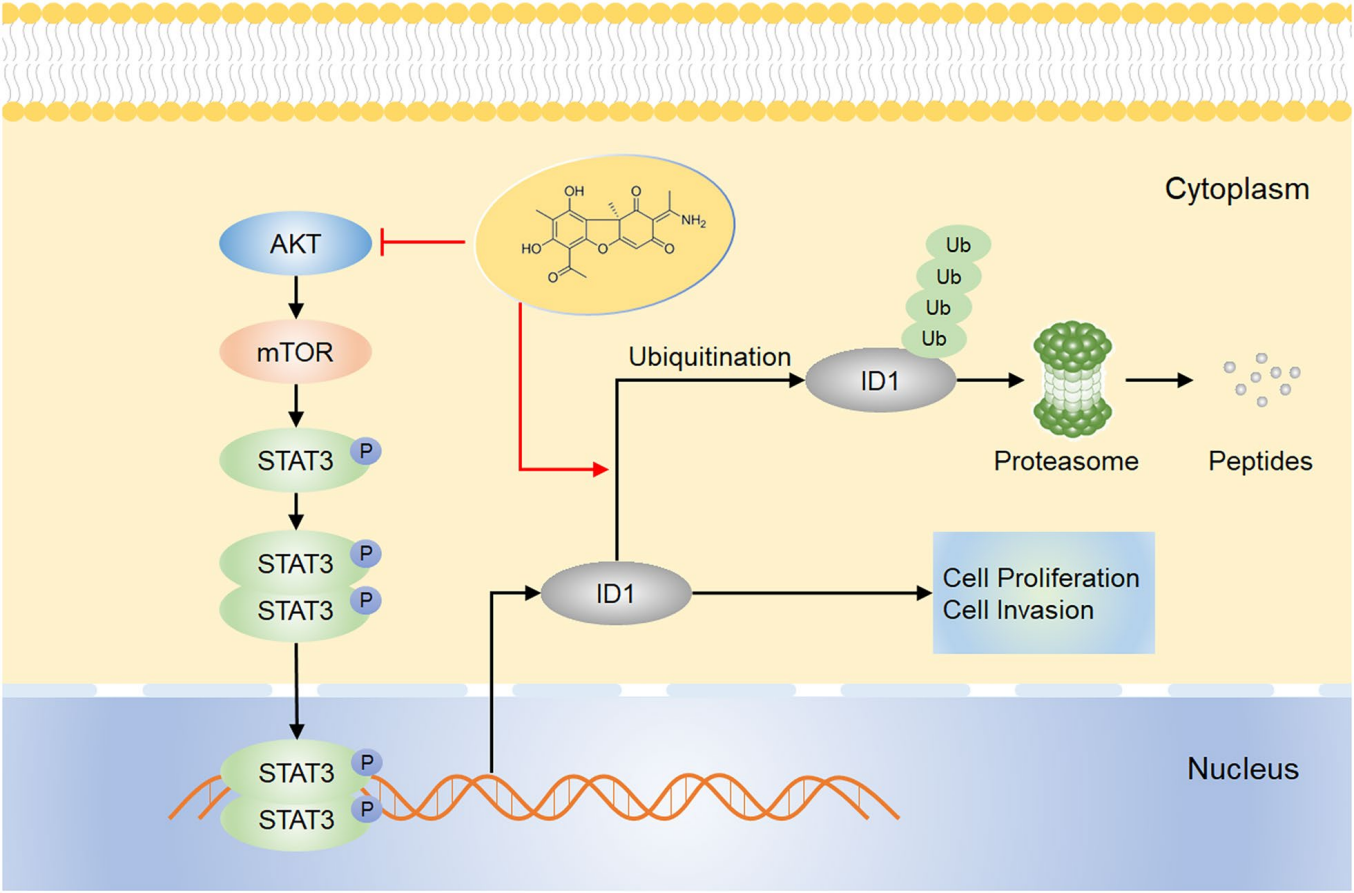
Sketch map showing mechanisms related to antihepatoma activity of usenamine A. Usenamine A suppressed the proliferative and metastatic potential of human HCC cells by repressing ID1 expression mediated by the down-regulation of AKT/mTOR/STAT3 signaling pathway and promotion of ubiquitin–proteasome-mediated degradation

In summary, we have demonstrated that usenamine A inhibited ID1 expression by downregulating AKT/mTOR/STAT3 signaling and fostering ubiquitin–proteasome-mediated ID1 degradation in human HCC cells (Fig. 9). In light of our discoveries, we propose that usenamine A holds significant promise as a prospective pharmaceutical agent for the management of HCC featuring aberrant AKT/mTOR/STAT3/ID1 signaling. Furthermore, targeting the AKT/mTOR/STAT3 signaling pathway can be considered an option for the treatment of HCC patients exhibiting elevated ID1 expression.

## Data Availability

All data generated or analysed during this study are included in this published article.

## Abbreviations

HCC: Hepatocellular carcinoma
ID1: Inhibitor of differentiation/DNA binding 1
STAT3: Signal transducer and activator of transcription 3
mTOR: Mechanistic target of rapamycin
ChIP: Chromatin immunoprecipitation
IHC analysis: Immunohistochemistry analysis
DMEM: Dulbecco’s Modified Eagle’s Medium
FBS: Fetal bovine serum
